# Trends in potentially avoidable hospitalizations for diabetes in Switzerland, 1998 to 2018: Data from multiple cross-sectional studies

**DOI:** 10.1016/j.heliyon.2024.e40466

**Published:** 2024-11-22

**Authors:** Ko Ko Maung, Charlène Mauron, Alexandre Gouveia, Pedro Marques-Vidal

**Affiliations:** a Department of Medicine, Internal Medicine, Lausanne University Hospital (CHUV) and University of Lausanne, Lausanne, Switzerland; b Centre for Primary Care and Public Health (Unisanté), University of Lausanne, Lausanne, Switzerland

**Keywords:** Potentially avoidable hospitalizations, Diabetes, Administrative data, Switzerland

## Abstract

**Background:**

Timely and appropriate outpatient care can prevent potentially avoidable hospitalizations (PAH) for diabetes. We analysed the trends, determinants, and consequences of PAH for diabetes in Switzerland over two decades.

**Methods:**

hospital discharge data for years 1998–2018 were used. PAH were defined according to the Organization for Economic Cooperation and Development criteria.

**Results:**

Overall, 90,853 admissions were considered as PAH, of which 25,376 (27.8 %), 9789 (10.8 %) and 55,688 (61.4 %) represented uncontrolled diabetes, short-term and long-term complications, respectively. The number of PAH increased from 2600 in 1998 to 5730 in 2018, mostly due to long-term complications. Compared to PAH for long-term complications, admissions for uncontrolled diabetes and short-term complications were more frequently women, in the younger age categories, more frequently non-Swiss, without health insurance, were more frequently admitted in an emergency setting, at the patient's initiative or via emergency services (ambulance, police), and had a lower comorbidity index. PAH for uncontrolled diabetes and short-term diabetic complications were more frequent in the non-German speaking regions. Patients with PAH for short-term diabetic complications were more frequently admitted to the intensive care unit, and their length of stay was shorter. In 2018, PAH for diabetes represented 56,255 days, corresponding to 155 hospital beds occupied yearly, and an estimated cost of 76 million CHF.

**Conclusion:**

In Switzerland, the number PAH for diabetes increased during the period 1998–2018. PAH types differ according to geographic region and to the patient's characteristics, and a sizable fraction is related to uncontrolled diabetes or likely preventable short-term complications.

## Highlights

• **What is already known?**In Switzerland, diabetes affects approximately 500,000 people and is on the rise.
• Providing timely and suitable outpatient care can help avoid unnecessary hospitalizations for diabetes.
• **What this study has found?**In Switzerland, the number PAH for diabetes increased from 2600 in 1998 to 5730 in 2018, mostly due to long-term complications.
• **What are the implications of the study?**Most PAH could be prevented, thus reducing cost and sparing health systems.

## Graphical abstract

**Figure:**
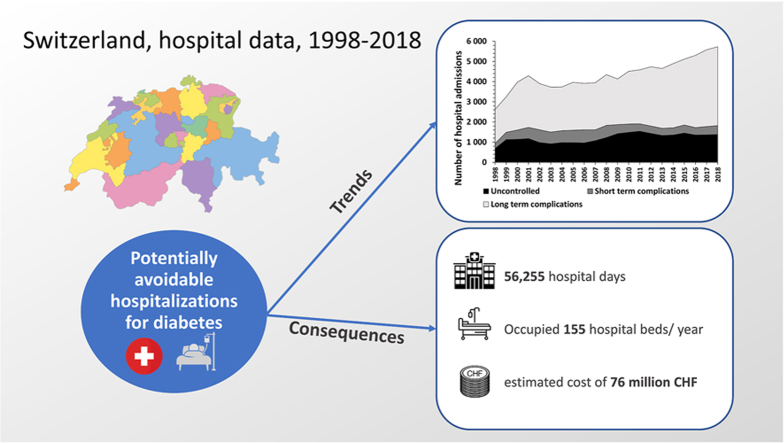

## 1 Introduction

Diabetes is one of the primary contributors to global mortality and disability irrespective of nationality, age, or gender. Projections of diabetes prevalence indicate a worrisome trend, with one study suggesting that the number of people with diabetes could reach a staggering 1.31 billion by 2050 [1]. Indeed, the prevalence of diabetes remains a significant concern, affecting approximately 422 million individuals worldwide, with 1.5 million diabetes-related deaths annually. Similarly, the prevalence of diabetes was around half a million in Switzerland [2], with an increasing trend [3], paralleled by an improvement in management. Still, over half of treated people with diabetes present with suboptimal control [4]. Diabetes is one of the ambulatory care sensitive conditions (ACSC), where timely and appropriate outpatient care has the potential to prevent the requirement for hospitalization or the escalation of complications [5]. A recent study suggested a substantially high number of potentially avoidable hospitalizations (PAH) for asthma and chronic obstructive airway diseases in Switzerland [6]. Whereas such a trend also occurs for diabetes has not been assessed.

Therefore, we aimed to evaluate the trends in PAH for diabetes in Switzerland for the period 1998 to 2018, and the factors associated with it. Our hypothesis was that, with better management of diabetes, the number of PAH for this disease is decreasing.

## 2 Methods

### 2.1 Data sources

Nationally representative data for Switzerland obtained from the Swiss Federal Office for Statistics, for the period 1998 to 2018 (contract number 200291). The data covers 98 % of public and private hospitals within Switzerland and includes all stays for each hospital.

### 2.2 Variables, data sources and measurements

The main cause for hospitalization and the comorbidities were coded using the 10th revision of the International Classification of Diseases (ICD-10) of the WHO. The data also contains information regarding gender, age (categorized into 5-year groups), administrative regions (26 Swiss cantons), date of admission (limited to month and year), decision of admission (i.e., patient's or doctor's initiative, others), type of admission (planned or emergency), type of room (infirmary, semi-private or private room), admission to intensive care unit (ICU), type of intervention (coded using the Swiss CHOP system) [7] and length of stay (LOS). For this analysis, we grouped the age categories into 10-year age groups and the 26 cantons into seven administrative regions, i.e., Leman, Mittelland, Northwest, Zurich, Eastern, Central, and Ticino (Supplemental Fig. 1).

Severity of disease was assessed using the Charlson's comorbidity index adapted to the Swiss population [8]. Briefly, this index considers 207 ICD-10 codes, which are weighted according to their severity. For example, presence of a secondary malignant neoplasm (ICD-10 code C78) will be given a weight of 6, while rheumatoid arthritis (ICD-10 code M05) will be given a weight of 1. The index was computed using data from the current hospitalization and patients were categorized into 0–1, 2–3 and 4+ score values.

### 2.3 Potentially avoidable hospitalizations and their consequences

The definition for a PAH for diabetes was obtained according to the international OECD Health Care Quality Indicators Project criteria [9] (Supplemental Table 1). PAH was then classified into long-term complications, short-term complications, and uncontrolled diabetes. Short-term complications were defined as not maternal or neonatal, that occur in people aged 15 years or older and are the result of an insulin deficiency; examples include coma or ketoacidosis. Long-term complications were defined as not maternal or neonatal, that occur in people aged 15 years or older, and include complications like renal, eye, or circulatory problems. Admissions for uncontrolled diabetes included inpatient admissions with the principal diagnosis code for uncontrolled diabetes [10].

The total number of days due to PAH was computed for each year. This number was then divided by 365 to obtain the number of beds that would be theoretically solely dedicated to PAH during that year.

Costs were computed for year 2018 using the Swiss Diagnosis-Related Group (DRG) system as indicated previously [6] and values were expressed in CHF (1 CHF = 1.02 € or 1.10 US$ as of May 8, 2024).

### 2.4 Inclusion and exclusion criteria

Hospitalizations related to adults (i.e., being at least in age group 20–24) and with a code for diabetes as the main cause of admission were eligible for analysis. Patients coming from outside of Switzerland or hospitalizations with missing covariates were excluded.

### 2.5 Statistical analysis

Analysis was conducted using Stata version 16.1 for Windows® (Stata Corp, College Station, TX, USA). Descriptive results were expressed as number of hospitalizations (percentage) or as median [interquartile range]. Between-group comparisons were performed using chi-square for categorical variables and Kruskal-Wallis test for continuous variables. The yearly trends in total and specific number of PAH categories were assessed using linear regression, and the results were expressed as slope and (95 % confidence interval). Multivariable analysis of the categorical variables associated with the different types of PAH was performed using multivariate (polytomous) logistic regression using long-term complications as reference category and results were expressed as relative risk ratio (equivalent to an odds ratio) and (95 % confidence interval). Multivariable analysis of LOS and costs was performed on log-transformed data using analysis of variance; values were back-transformed and expressed as mean ± standard error. Statistical significance was considered for a two-sided test with p < 0.05.

## 3 Results

### 3.1 Inclusion and exclusion criteria

According to the definition of PAH of diabetes by the OECD, all hospital admissions with a principal diagnosis code of diabetes are eligible. Cases where the patient died in hospital during the admission, cases resulting from a transfer from another acute care institution (transfers-in), obstetric hospitalisations, childbirth, and puerperium codes in any field and cases that are same day/day only admissions were excluded (Supplementary Table 1).

### 3.2 Trends of potentially avoidable hospitalizations

**Fig. 1:**
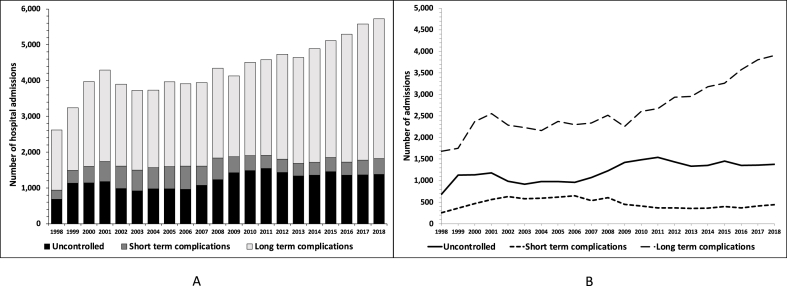
Trends in the number of hospitalizations for diabetes and percentage of potentially avoidable hospitalizations, Switzerland, 1998–2018.

Fig. 1A and **B** depict the trend of PAH for diabetes from 1998 to 2018. The number of PAH for diabetes increased almost twofold from approximately 2600 in 1998 to 5730 in 2018. Notably, long-term diabetic complications demonstrated the majority of the for diabetes and the most substantial increase (from 1679 (64 %) in 1998 to 3903 (68 %) in 2018) among the various categories of PAH for diabetes whereas the percentage of PAH for uncontrolled and short-term diabetic complications decreased. Overall, the number of PAH uncontrolled diabetic complications increased on average by 28 per year (17–40), those of short-term diabetic complications did not show a significant trend (p = 0.162), those of long-term diabetic complications increased by 89 per year (70–109) and the overall number of PAH increased by 112 per year (89–135).

#### 3.2.1 General characteristics of potentially avoidable hospitalizations

Total admissions for diabetes represented between 0.3 and 0.5 % of all hospital admissions in Switzerland, while admissions for PAH represented an even smaller percentage, ranging between 0.13 and 0.21 %. Overall, 90,853 admissions were considered as PAH, of which 25,376 (27.8 %), 9789 (10.8 %) and 55,688 (61.4 %) represented uncontrolled diabetes, short-term and long-term complications, respectively. The results of the bivariate analysis of general characteristics of the PAH compared to non-PAH are summarised in Table 1. Compared to PAH for long-term diabetic complications, admissions with PAH for uncontrolled diabetes and short-term diabetic complications were more frequently women, in the younger age categories, more frequently non-Swiss, without health insurance, were more frequently admitted in an emergency setting, at the patient's initiative or via emergency services (ambulance, police), and had a lower comorbidity index. PAH for uncontrolled diabetes and short-term diabetic complications were more frequent in the non-German speaking regions, Leman and Tessin.

**Table 1:** Characteristics of potentially avoidable hospitalizations for diabetes, Switzerland, 1998–2018.

	Bivariate analysis			Multivariate analysis			
	Uncontrolled	Short-term complications	Long-term complications	Uncontrolled	p-value	Short-term complications	p-value
Sample size	25,376	9789	55,688				
Women	11510 (45.4)	4587 (46.9)	20979 (37.7)	1.17 (1.12–1.22)	<0.001	1.36 (1.29–1.43)	<0.001
Age groups							
[20–30[	1299 (5.1)	1320 (13.5)	770 (1.4)	1 (ref.)		1 (ref.)	
[30–40[	1659 (6.5)	1018 (10.4)	1603 (2.9)	0.97 (0.83–1.12)	0.658	0.56 (0.48–0.65)	<0.001
[40–50[	3292 (13.0)	1275 (13.0)	3969 (7.1)	1.14 (1.00–1.31)	0.055	0.41 (0.36–0.48)	<0.001
[50–60[	4949 (19.5)	1580 (16.1)	8829 (15.9)	1.05 (0.92–1.19)	0.488	0.29 (0.26–0.34)	<0.001
[60–70[	4859 (19.2)	1476 (15.1)	13390 (24.0)	0.95 (0.83–1.08)	0.410	0.23 (0.20–0.26)	<0.001
[70–80[	5018 (19.8)	1753 (17.9)	15802 (28.4)	0.99 (0.87–1.12)	0.869	0.22 (0.19–0.25)	<0.001
[80–90[	3741 (14.7)	1212 (12.4)	10123 (18.2)	1.21 (1.06–1.37)	0.005	0.22 (0.19–0.26)	<0.001
[90+	559 (2.2)	155 (1.6)	1202 (2.2)	1.32 (1.10–1.57)	0.002	0.21 (0.17–0.26)	<0.001
Non-Swiss	6184 (24.4)	1998 (20.4)	9877 (17.7)	1.19 (1.13–1.26)	<0.001	0.90 (0.84–0.96)	0.002
Region							
Leman	4906 (19.3)	1935 (19.8)	8117 (14.6)	1.46 (1.36–1.56)	<0.001	1.18 (1.08–1.28)	<0.001
Mittelland	4453 (17.6)	2011 (20.5)	11591 (20.8)	1 (ref.)		1 (ref.)	
Northwest	4266 (16.8)	1528 (15.6)	10007 (18.0)	0.90 (0.84–0.96)	0.002	0.76 (0.69–0.83)	<0.001
Zurich	3618 (14.3)	1425 (14.6)	10018 (18.0)	0.95 (0.89–1.02)	0.139	0.72 (0.66–0.79)	<0.001
Eastern	3639 (14.3)	1045 (10.7)	7700 (13.8)	0.98 (0.91–1.05)	0.530	0.63 (0.58–0.70)	<0.001
Central	2024 (8.0)	905 (9.3)	4668 (8.4)	0.95 (0.87–1.03)	0.216	0.99 (0.89–1.10)	0.840
Tessin	2470 (9.7)	940 (9.6)	3587 (6.4)	1.83 (1.69–1.99)	<0.001	2.03 (1.83–2.24)	<0.001
No insurance	682 (2.7)	353 (3.6)	1205 (2.2)	0.72 (0.63–0.83)	<0.001	1.06 (0.91–1.24)	0.445
Emergency	16246 (64.0)	8236 (84.1)	31068 (55.8)	1.61 (1.54–1.69)	<0.001	4.29 (3.99–4.60)	<0.001
Admission							
Patient	5203 (20.5)	2585 (26.4)	9576 (17.2)	0.86 (0.81–0.91)	<0.001	1.41 (1.32–1.51)	<0.001
Ambulance	2776 (10.9)	2720 (27.8)	7081 (12.7)	0.81 (0.76–0.86)	<0.001	2.90 (2.71–3.11)	<0.001
Doctor	17107 (67.4)	4389 (44.8)	38478 (69.1)	1 (ref.)		1 (ref.)	
Other	290 (1.1)	95 (1.0)	553 (1.0)	0.97 (0.79–1.18)	0.746	1.03 (0.79–1.34)	0.842
Charlson index							
0–1	18191 (71.7)	6335 (64.7)	2721 (4.9)	1 (ref.)		1 (ref.)	
2–3	5343 (21.1)	2139 (21.9)	34029 (61.1)	0.02 (0.02–0.02)	<0.001	0.03 (0.03–0.03)	<0.001
4+	1842 (7.3)	1315 (13.4)	18938 (34.0)	0.01 (0.01–0.01)	<0.001	0.04 (0.04–0.04)	<0.001

Ref: Reference. Results are expressed as number of admissions (column percentage) for bivariate analysis and expressed as relative risk ratios and (95 % confidence interval), using long-term complications as reference for the multivariate analysis. Statistical comparisons performed using chi-square and multinomial (polytomous) logistic regression. All comparisons of the bivariate analysis are significant at p < 0.001.

The results of the multivariable analysis are provided in Table 1; long-term diabetic complications were used as a reference. Women, older patients, non-Swiss, people living in Leman or Tessin regions, or coming to the hospital via the emergency department had a higher likelihood of being admitted for uncontrolled diabetes, while people living in the Northwest region of Switzerland, people without insurance, coming to the hospital via the patient's initiative or ambulance, or patients with a high Charlson index had a lower likelihood of being admitted for uncontrolled diabetes. Women, people living in Leman or Tessin regions, people coming to the hospital via the emergency department, via the patient's initiative or ambulance had a higher likelihood of being admitted for short-term complications of diabetes, while being older than 30, people living in Northwest, Zurich, and Eastern regions, or patients with a high Charlson index had a lower likelihood of being admitted for short-term complications for diabetes.

### 3.3 Consequence of potentially avoidable hospitalizations

The consequences of the different PAH for diabetes are provided in Table 2. Compared to PAH for long-term diabetic complications, PAH for uncontrolled diabetes and short-term diabetic complications were more frequently admitted to the ICU, were more frequently discharged home, and their LOS was shorter, and this was confirmed after multivariable adjustment.

**Table 2:** Consequences of potentially avoidable hospitalizations for diabetes, Switzerland, 1998–2018.

	Uncontrolled	Short-term complications	Long-term complications
N	25,376	9789	55,688
Type of hospital stay (%)			
Infirmary	21,204 (83.6)	8250 (84.3)	45,489 (81.7)
Semi-private	2587 (10.2)	1035 (10.6)	6771 (12.2)
Private	1585 (6.3)	504 (5.2)	3428 (6.2)
Intensive care unit stay (%)	1384 (5.5)	3096 (31.7)	2667 (4.8)
Destination at discharge (%)			
Home	22,560 (88.9)	8195 (83.7)	46,627 (83.7)
Medical home	1166 (4.6)	505 (5.2)	3761 (6.8)
Other	1650 (6.5)	1089 (11.1)	5300 (9.5)
Length of stay (days)			
Bivariate	8 [5–12]	8 [5–13]	9 [5–16]
Multivariable	8 ± 1	7.8 ± 1	8.5 ± 1
Estimated cost (CHF)			
Bivariate	6901 [6219–9135]	10,181 [9225–10,377]	10,377 [8770–17,090]
Multivariable	9000 ± 1	12,298 ± 1	12,356 ± 1
Total cost 2018^a^	10.0	5.4	60.7

Results are expressed as median [interquartile range] or as multivariable adjusted mean standard error. Bivariate analyses conducted using Kruskal-Wallis test. Multivariable analyses conducted using analysis of variance on log-transformed data, adjusting for gender, age categories, nationality, administrative region, insurance, type of admission, ICU stay and categories of Charlson index. Multivariable results were exponentiated for presentation. All comparisons are significant at <0.001.

a in million CHF.

**Fig. 2:**
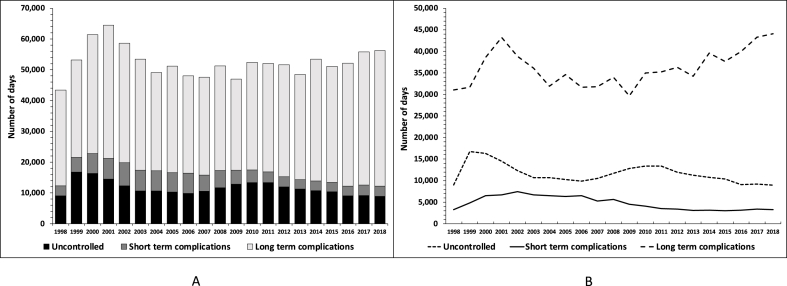
Trends in the number of hospital days corresponding to potentially avoidable hospitalizations for diabetes, Switzerland, 1998–2018. Panel A, total number of hospital days for all potentially avoidable hospitalizations for diabetes. Panel B, number of hospital days for potentially avoidable hospitalizations long-term complications, short-term complications, and uncontrolled diabetes.

**Fig. 3:**
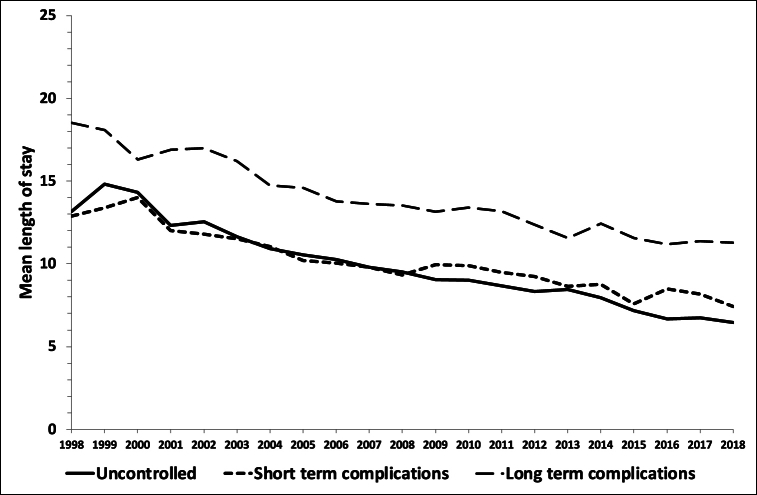
Trends in average length of stay of potentially avoidable hospitalizations for diabetes, Switzerland, 1998–2018.

The number of hospital days related to PAH for diabetes from 1998 to 2018 is described in Fig. 2A and **B**. There was a sharp rise from 43,366 days in 1998 to 64,412 days in 2001, followed by a downward trend for 4 years, after which a slow upward trend occurred, to reach 56,255 days in 2018. This last value corresponds to 155 hospital beds fully occupied by PAH for diabetes during a whole year. The average LOS for each PAH showed a consistent decrease, being almost halved between 1998 and 2018 for uncontrolled and short-term complications (Fig. 3).

On bivariate analysis, the median costs of a PAH for short-term diabetic complications and long-term complications were considerably higher than for a PAH for uncontrolled diabetes, and similar findings were obtained after multivariable adjustment. in 2018, the estimated total cost for PAH for diabetes amounted to approximately 10 million CHF for short-term diabetic complications, 60.7 million CHF for long-term diabetic complications, and 5.4 million CHF for uncontrolled diabetes.

## 4 Discussion

In Switzerland, the number of PAH for diabetes increased during the period 1998–2018. However, relative to all hospital admissions in Switzerland, PHA for diabetes represented a very minute percentage of admissions. Types of PAH differ according to geographic region and to the patient's characteristics, and a sizable fraction is related to uncontrolled diabetes or likely preventable short-term complications.

PAH due to ACSC have been widely used as a tool for the measurement of the accessibility and effectiveness of primary care services within a given region. By analysing rates, trends, determinants, and costs of PAH, healthcare stakeholders can assess the quality of healthcare and gain essential insights for strategic resource planning, particularly in addressing the prevention and management of chronic diseases at the primary care level.

### 4.1 Trends of potentially avoidable hospitalizations

Our study showed that the total number of PAH in Switzerland increased almost twofold from 1998 to 2018. This increase is in agreement with the studies in Asian countries such as Japan, Singapore, Hong Kong, and Beijing, where PAH rates increased [11], but in contrast to other studies in European countries where PAH rates decreased [12,13]; different definitions of PAH could be an explanation for the different results. Nevertheless, according to the recent data from OECD, when compared to other European countries, Switzerland has maintained the relatively low prevalence of PAH [14]. This could probably be related to one of the highest rates of medically equipped general practitioners in Europe [10]. Indeed, the healthcare system in Switzerland is regarded as one of the best in the world.

#### 4.1.1 General characteristics of potentially avoidable hospitalizations

According to the OECD, all non-maternal/non-neonatal hospital admissions with a principal diagnosis code of diabetes are considered potentially avoidable (Supplementary Table 1). However, uncontrolled diabetes and short-term diabetic complications are theoretically assumed to be more preventable with timely and appropriate outpatient care compared to long-term diabetic complications. Indeed, the latter occurred in elderly patients with a high Charlson index, suggesting that PAH for long-term diabetic complications might be due to the increase in the elderly population [15], associated with increased polypharmacy and corresponding drug-drug interactions [16] needed to manage multiple comorbidities in those patients.

PAH for short-term diabetic complications included younger individuals with fewer comorbidities (i.e. a low Charlson Index), a finding also reported in a Canadian study [17]. This might probably be related to poor compliance with anti-diabetic treatment in younger diabetic patients [18,19] or their inability to manage hypoglycaemic or hyperglycaemic events [20]. Indeed, over 84 % of the PAH for short-term diabetic complications in Switzerland were admitted via the emergency ward, were more likely to be decided either by patient's initiative or ambulance or police, and almost one-third required ICU. Moreover, people who were non-Swiss or without health insurance had a higher PAH of short-term diabetic complications rate, a finding likely to be associated with lower socio-economic status, a risk factor for PAH for diabetes [20]. Recent consensus report on short-term complications such as recurrent episodes of diabetic ketoacidosis were that they represent majority of DKA-related readmissions. It was also suggested that these episodes are more common in younger people with diabetes possibly related to psychological disorders such as depression, substance use and poor socioeconomic status [21]. On the other hand, short-term diabetes complications such as hypoglycaemia increase considerably after the age of 65+ [22]. Hence, it is likely that, with the ageing of the population, more of such events will occur in the future. Overall, our results indicate that most PAH for short-term diabetic complications are likely due to the patients' mismanagement of their disease, and that adequate education could eventually reduce PAH and thus their costs.

“In general, coexisting multiple comorbidities, metastatic cancers, alcohol abuse, drug abuse poor socioeconomic status, despite the universal health coverage, and underlying serious psychiatric conditions are found to be the significant risk factors of PAH for diabetes [23,24].

### 4.2 Consequence of potentially avoidable hospitalizations

Almost one-third of patients with PAH for short-term diabetic complications were admitted to ICU, versus 5 % for uncontrolled and long-term diabetic complications. The LOS of PAH for short-term diabetic complications was also shorter, suggesting that many admissions occurred for the management of acute conditions such as hyper or hypoglycaemia, conditions easily preventable if the patients are instructed how to act in such conditions.

The number of hospital beds needed for PAH for diabetes increased, but at a smaller pace than the number of PAH. This was due to a reduction in LOS with time, suggestive of an improvement in the in-hospital management of admissions for diabetes, as reported elsewhere [25,26]. In 2018, PAH for diabetes represented 155 beds fully occupied per year, or 0.4 % of the 38,051 hospital beds for Switzerland [27]. Although this value might be small, still it could be reduced with adequate measures.

The total cost of PAH was around 76 million CHF in 2018. This value might be considered minimal compared to the global health expenditures in Switzerland (86,300 million CHF in 2021) [28]. Still, the individual cost of a PAH for diabetes was relatively high. For PAH for short-term complications, the high cost is due to the high use of ICU because of hyper or hypoglycaemia events; hence, better management of diabetes could lead to significant savings. On the other hand, from the macroeconomic perspective, almost 80 % of the estimated total cost of PAH was due to long-term complications. Several studies showed that lower limb amputation, a major long-term complication of diabetes, has been decreasing in most high-income countries [29,30], likely due to improvement in primary care, frequent monitoring, and regular follow up of the people with diabetes. Overall, our results suggest that, although the total cost of PAH for diabetes is relatively low, it could be decreased via better management of diabetes by the patients and their caregivers [31].

### 4.3 Study strengths and limitations

The major strength of this study it that it was conducted in a nationwide setting, using data covering over 95 % of all Swiss hospitals. The data was collected using a standardized procedure throughout time, thus allowing assessing trends.

This study also has some limitations. Firstly, costs, were estimated and not directly obtained from the hospitals, so the cost, might be underestimated as they correspond to the amounts reimbursed by the health insurance companies and not the true costs. Amputation imposes a considerable economic burden on health systems [32,33]. In Switzerland, although the cost of surgery and invasive procedures is usually reimbursed according to the CHOP codes indicated by the hospital, it is possible that those costs be underestimated. Further, the indirect costs related to productivity losses or informal care were not considered. Still, they provide a minimum value for the PAH-related costs. Secondly, our results were obtained in a wealthy country with a very good healthcare system and might not apply to countries with a less developed health system. Thirdly, potential confounders on PAH such as socioeconomic, medication and body mass index were not available in the data set; this might lead to residual confounding resulting in possible overestimation or underestimating of the outcome. Fourthly, no information regarding the cause of admission (i.e. if for amputation or other conditions such as ketosis) was available.

## 5 Conclusion

In Switzerland, the number of PAH for diabetes increased during the period 1998–2018. PAH types differ according to geographic region and to the patient's characteristics, and a sizable fraction is related to uncontrolled diabetes or likely preventable short-term complications. Costs of PAH from uncontrolled and short-term complications could be reduced via an adequate management of diabetes.

## CRediT authorship contribution statement

**Ko Ko Maung:** Writing – original draft, Visualization, Investigation, Formal analysis. **Charlène Mauron:** Writing – review & editing. **Alexandre Gouveia:** Writing – review & editing, Methodology, Conceptualization. **Pedro Marques-Vidal:** Writing – review & editing, Validation, Supervision, Data curation, Conceptualization.

## Ethical statement

The data of the Swiss Federal Office of Statistics is available for research purposes and therefore neither specific individual consent nor authorization from an Ethics Committee was needed. Data that is collected routinely in the Medical Statistics of Hospitals are de-identified.

## Data sharing statement

Due to legal constraints, sharing of the data is not allowed. People interested in obtaining the data should contact the Swiss Federal Office of Statistics (www.bfs.admin.ch) for further information.

## Funding

This research received no specific grant from any funding agency in the public, commercial or not-for-profit sectors.

## Declaration of competing interest

The authors declare that they have no known competing financial interests or personal relationships that could have appeared to influence the work reported in this paper.
