# Supplementary material for: Trends in potentially avoidable hospitalizations for diabetes in Switzerland, 1998 to 2018: Data from multiple cross-sectional studies
Source: Heliyon. 2024 Nov 22;10(23):e40466. doi: 10.1016/j.heliyon.2024.e40466 (PMC11647795; doi:10.1016/j.heliyon.2024.e40466)
Supplement: Multimedia component 1 [file mmc1.docx]

**Supplementary table 1:** ICD-10-WHO codes used to create outcome measures

Source: OECD, Definitions of Health Care Quality Indicators, 2012-2013 HCQI data collection

| **Uncontrolled diabetes** |
| --- |
| E109 insulin-dependent diabetes mellitus without complications  E119 non-insulin-dependent diabetes mellitus without complications  E139 other specified diabetes mellitus without complications  E149 unspecified diabetes mellitus without complications |

| **Diabetes long-term complications** |
| --- |
| E102 insulin-dependent dm with renal c omplications  E103 insulin-dependent dm with ophthalmic complications  E104 insulin-dependent dm with neurological complications  E105 insulin-dependent dm with peripheral circulatory complications  E106 insulin-dependent dm with other specified complications  E107 insulin-dependent dm with multiple complications  E108 insulin-dependent dm with unspecified complications  E112 non-insulin-dependent dm with renal complications  E113 non-insulin-dependent dm with ophthalmic complications  E114 non-insulin-dependent dm with neurological complications  E115 non-insulin-dependent dm with peripheral circulatory complications  E116 non-insulin-dependent dm with other specified complications  E117 non-insulin-dependent dm with multiple complications  E118 non-insulin-dependent dm with unspecified complications  E132 other specified dm with renal complications E133 other specified dm with ophthalmic complications  E134 other specified dm with neurological complications  E135 other specified dm with peripheral circulatory complications  E136 other specified dm with other specified complications  E137 other specified dm with multiple complications E138 other specified dm with unspecified complications  E142 unspecified dm with renal complications E143 unspecified dm with ophthalmic complications E144 unspecified dm with neurological complications  E145 unspecified dm with peripheral circulatory complications  E146 unspecified dm with other specified complications  E147 unspecified dm with multiple complications E148 unspecified dm with unspecified complications |

| **Diabetes short-term complications** |
| --- |
| E100 insulin-dependent diabetes mellitus with coma  E101 insulin-dependent diabetes mellitus with ketoacidosis  E110 non-insulin-dependent diabetes mellitus with coma  E111 non-insulin-dependent diabetes mellitus with ketoacidosis  E130 other specified diabetes mellitus with coma E131 other specified diabetes mellitus with ketoacidosis  E140 unspecified diabetes mellitus with coma E141 unspecified diabetes mellitus with  ketoacidosis |
