# Supplementary material for: Trends in potentially avoidable hospitalizations for diabetes in Switzerland, 1998 to 2018: Data from multiple cross-sectional studies
Source: Heliyon. 2024 Nov 22;10(23):e40466. doi: 10.1016/j.heliyon.2024.e40466 (PMC11647795; doi:10.1016/j.heliyon.2024.e40466)
Supplement: Multimedia component 2 [file mmc2.pptx]

## Slide 1
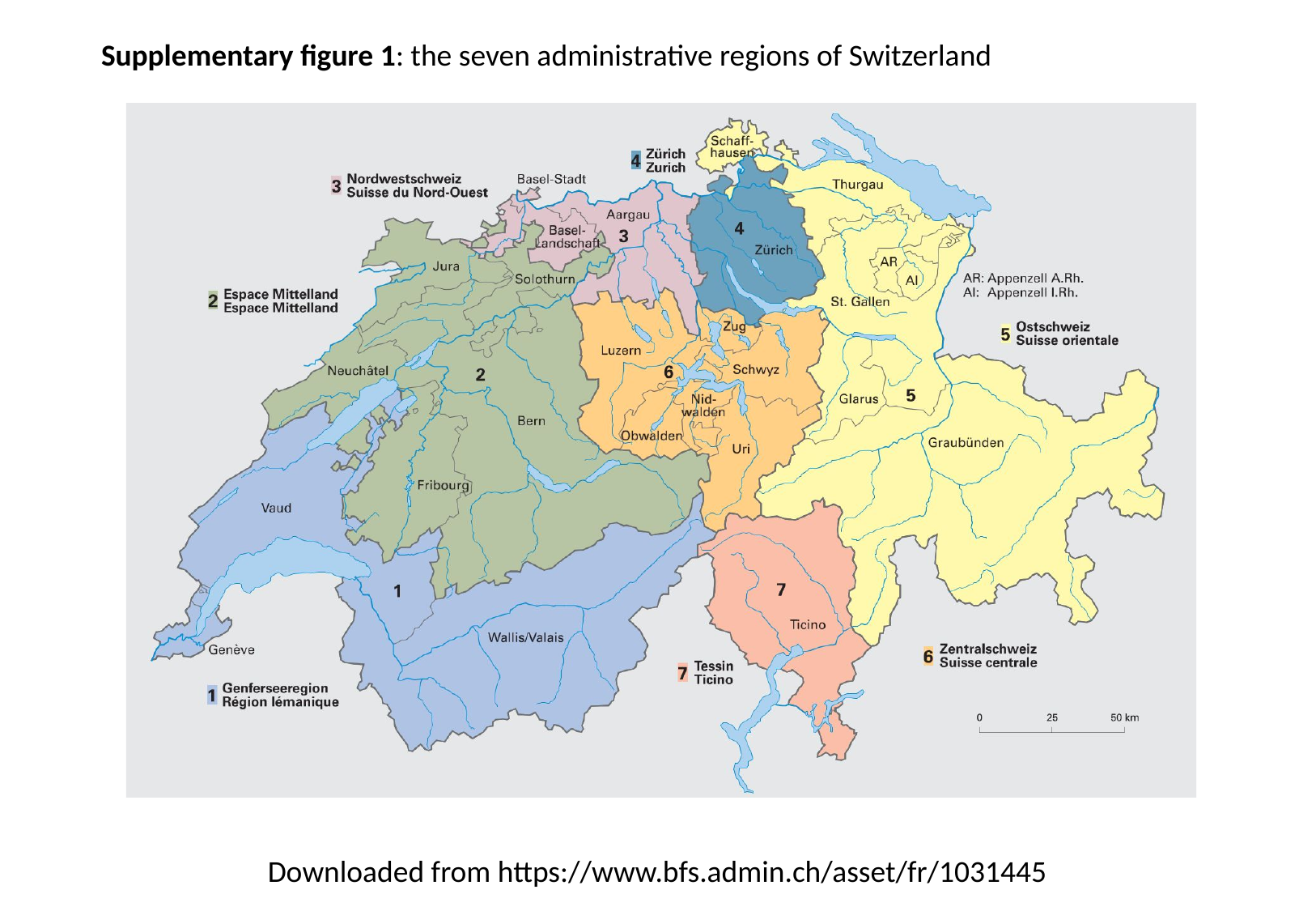

Supplementary figure 1: the seven administrative regions of Switzerland
Downloaded from https://www.bfs.admin.ch/asset/fr/1031445
